# Supplementary material for: Heat adaptation of phage T7 under an extended genetic code
Source: Virus Evol. 2021 Dec 1;7(2):veab100. doi: 10.1093/ve/veab100 (PMC8923235; doi:10.1093/ve/veab100)
Supplement: veab100_Supp [file veab100_supp.zip › Sequencing_Analysis.zip!/Sequencing_Analysis/S1/evidence/RA_36.html]

BRESEQ :: Evidence


| Read alignment evidence... | | | | | | | | | | | |
| --- | --- | --- | --- | --- | --- | --- | --- | --- | --- | --- | --- |
|  | seq id | position | | ref | new | freq | score (cons/poly) | reads | annotation | genes | product |
| \* | NC\_001604.1 | 39,930 | 1 | . | C | 44.4% | 3.8 / 13.5 | 9 | intergenic (–/–) | *–/–* | –/– |
| Reads supporting (aligned to +/- strand):  **ref** base . (0/5);  **new** base C (4/0);  **total** (4/5) | | | | | | | | | | | |
| Fisher's exact test for biased strand distribution *p*-value = 7.94e-03 | | | | | | | | | | | |
| Kolmogorov-Smirnov test that lower quality scores support variant *p*-value = 1.00e+00 | | | | | | | | | | | |

Only 100 of 106 total aligned reads displayed.  

`ATACCCATAAAGAGCCTCTAATGGTCTATCCTAAGGTCTATACCTAAAGATAGGCCATCCTATCA‑GTGTCACCTAAAGAGGGTCTTAGAGAGGGCCTATGGAGTTCCTATAGGGTCCTTTAAAATATACCATAAAAATCTGAGTGACTATCTCACAGTGTACGGACCTAAAGTTCCCCCATAGGGGGTACCTAAAGCCCAGCCAATCACCTAAAGTCAACCTTCGGTTGACCTTGAGGGTTCCCTAAGGGTTGGGGATGACCCTTGGGTTTGTCTTTGGGTGTTACCTTGAGTGTCTCTCTG‑TGTCCCT‑‑‑‑‑‑‑‑‑‑‑‑‑‑‑‑‑‑‑‑‑‑‑‑‑‑  >  NC_001604.1/39629‑39937`  

                                                                                                                                                                                                                                                                                                               `|`                                   
`cagcccATAAAGGGCCTCTAATGGTCTATCCTAAGGTCTATACCTAAAGATAGGCCATCCTATCA‑GTGTCACCTAAAGAGGGTCTTAGAGAGGGCCTATGGAGTTCCTATAGGGTCCTTTAAAATATACCATAAAAATCTGAGTGACTATCTCACAGTGTACGGACCTAAAGTTCCCCCATA‑GGGGTACCTAAAGCCCAGCCAATCACCTAAAGTCAACCTTCGGTTGACCTTGAGGGTTCCCTAAGGGTTGGGGATGACCCTTGGGTTTGTCTTTGGGTGTTACCTTGAGTGTCTCTCtg                                    <  1:407247/298‑1 (MQ=255)`  
`ataCCCATAAAGAGCCTCTAATGGTCTATCCTAAGGTCTATACCTAAAGATAGGCCATCCTATCA‑GTGTCACCT‑AAGAGGGTCTTAGAGAGGGCCTATGGAGTTCCTATAGGGTCCTTTAAAATATACCATAAAAATCTGAGTGACTATCTCACAGTGTACGGACCTAAAGTTCCCCCATAGGGGGTACCTAAAGCCCAGCCAATCACCTAAAGTCAACCTTCGGTTGACCTTGAGGGTTCCCTAAGGGTTGGGGATGACCCTTGGGTTTGTCTTTGGGTGTTACCTTGAGTGTCTCTCtg                                    <  1:297721/301‑1 (MQ=255)`  
 `tgtgtataagagacagctAATGGTCTATCCTAAGGTCTATACCTAAAGATAGGCCATCCTATCA‑GTGTCACCTAAAGAGGGTCTTAGAGAGGGCCTATGGAGTTCCTATAGGGTCCTTTAAAATATACCATAAAAATCTGAGTGACTATCTCACAGTGTACGGACCTAAAGTTCCCCCATAGGGGGTACCTAAAGCCCAGCCAATCACCTAAAGTCAACCTTCGGTTGACCTTGAGGGTTCCCTAAGGGTTGGGGATGACCCTTGGGTTTGTCTTTGGGTGTTACCTTGAGTGTCTCTCtg                                    <  1:488399/285‑1 (MQ=255)`  
 `tgtgtataagagacagctAATGGTCTATCCTAAGGTCTATACCTAAAGATAGGCCATCCTATCA‑GTGTCACCTAAAGAGGGTCTTAGAGAGGGCCTATGGAGTTCCTATAGGGTCCTTTAAAATATACCATAAAAATCTGAGTGACTATCTCACAGTGTACGGACCTAAAGTTCCCCCATAGGGGGTACCTAAAGCCCAGCCAATCACCTAAAGTCAACCTTCGGTTGACCTTGAGGGTTCCCTAAGGGTTGGGGATGACCCTTGGGTTTGTCTTTGGGTGTTACCTTGAGTGTCTCTCtg                                    <  1:611559/285‑1 (MQ=255)`  
 `tgtgattaagggaaggctAATGGTCTATCCTAAGGTCTATACCTAAAGATAGGCCATCCTATCA‑GTGTCACCTAAAGAGGGTCTTAGAGAGGTCCTATGGAGTTCCTATAGGGTCCTTTAAAATATACCATAAAAATCTGAGTGACTATCTCACAGTGTACGGACCTAAAGTTCCCCCATAGGGGGTACCTAAAGCCCAGCCAATCACCTAAAGTCAACCTTCGGTTGACCTTGAGGGTTCCCTAAGGGTTGGGGATGACCCTTGGGTTTGTCTTTGGGTGTTACCTTGAGTGTCTCTCtg                                    <  1:156626/285‑1 (MQ=255)`  
 `tgtataagagtcagctctAATGGTCTATCCTAAGGTCTATACCTAAAGATAGGCCATCCTATCA‑GTGTCACCTAAAGAGGGTCTTAGAGAGGGCCTATGGAGTTCCTATAGGGTCCTTTAAAATATACCATAAAAATCTGAGTGACTATCTCACAGTGTACGGACCTAAAGTTCCCCCATAGGGGGTACCTAAAGCCCAGCCAATCACCTAAAGTCAACCTTCGGTTGACCTTGAGGGTTCCCTAAGGGTTGGGGATGACCCTTGGGTTTGTCTTTGGGTGTTACCTTGAGTGTCTCTCtg                                    <  1:411509/287‑1 (MQ=255)`  
 `tgtataagagacagctctAATGGTCTATCCTAAGGTCTATACCTAAAGATAGGCCATCCTATCA‑GTGTCACCTAAAGAGGGTCTTAGAGAGGTCCTATGGAGTTCCTATAGGGTCCTTTAAAATATACCATAAAAATCTGAGTGACTATCTCACAGTGTACGGACCTAAAGTTCCCCCATAGGGGTTACCTAAAGCCCAGCCAATCACCTAAAGTCAACCTTCGGTTGACCTTGAGGGTTCCCTAAGGGTTGGGGATGACCCTTGGGTTTGTCTTTGGGTGTTACCTTGAGTGTCTCTCtg                                    <  1:373353/287‑1 (MQ=255)`  
 `tgtataagagacagctctAATGGTCTATCCTAAGGTCTATACCTAAAGATAGGCCATCCTATCA‑GTGTCACCTAAAGAGGGTCTTAGAGAGGGCCTATGGAGTTCCTATAGGGTCCTTTAAAATATACCATAAAAATCTGAGTGACTATCTCACAGTGTACGGACCTAAAGTTCCCCCATAGGGGGTACCTAAAGCCCAGCCAATCACCTAAAGTCAACCTTCGGTTGACCTTGAGGGTTCCCTAAGGGTTGGGGATGACCCTTGGGTTTGTCTTTGGGTGTTACCTTGAGTGTCTCTCtg                                    <  1:512596/287‑1 (MQ=255)`  
 `tgtataagagacagctctAATGGTCTATCCTAAGGTCTATACCTAAAGATAGGCCATCCTATCA‑GGGTCACCTAAAGAGGGTCTTAGAGAGGGCCTATGGAGTTCCTATAGGGTCCTTTAAAATATACCATAAAAATCTGAGTGACTATCTCACAGTGTACGGACCTAAAGTTCCCCCATAGGGGGTACCTAAAGCCCAGCCAATCACCTAAAGTCAACCTTCGGTTGACCTTGAGGGTTCCCTAAGGGTTGGGGATGACCCTTGGGTTTGTCTTTGGGTGTTACCTTGAGTGTCTCTCtg                                    <  1:513004/287‑1 (MQ=255)`  
 `tgtataagagacagctctAATGGTCTATCCGAAGGTCTATACCTAAAGATAGGCCATCCTATCA‑GTGTCACCTAAAGAGGGTCTTAGAGAGGGCCTATGGAGTTCCTATAGGGTCCTTTAAAATATACCATAAAAATCTGAGTGACTATCTCACAGTGTACGGACCTAAAGTTCCCCCATAGGGGGTACCTAAAGCCCAGCCAATCACCTAAAGTCAACCTTCGGTTGACCTTGAGGGTTCCCTAAGGGTTGGGGATGACCCTTGGGTTTGTCTTTGGGTGTTACCTTGAGTGTCTCTCtg                                    <  1:228321/287‑1 (MQ=255)`  
 `tgtataagagacagctctAATGGGCTATCCTAAGGTCTATACCTAAAGATAGGCCATCCTAGCA‑GTGTCACCTAAAGAGGGTCTTAGAGAGGGCCTATGGAGTTCCTATAGGGTCCTTTAAAATATACCATAAAAATCTGAGTGACTATCTCACAGTGTACGGACCTAAAGTTCCCCCATAGGGGGTACCTAAAGCCCAGCCAATCACCTAAAGTCAACCTTCGGTTGACCTTGAGGGTTCCCTAAGGGTTGGGGATGACCCTTGGGTTTGTCTTTGGGTGTTACCTTGAGTGTCTCTCtg                                    <  1:542612/287‑1 (MQ=255)`  
 `tataagagacaggCCTCTAATGGTCTATCCTAAGGTCTATACCTAAAGATAGGCCATCCTATCA‑GTGTCACCTAAAGAGGGTCTTAGAGAGGGCCTATGGAGTTCCTATAGGGTCCTTTAAAATATACCATAAAAATCTGAGTGACTATCTCACAGTGTACGGACCTAAAGTTCCCCCATAGGGGGTACCTAAAGCCCAGCCAATCACCTAAAGTCAACCTTCGGTTGACCTTGAGGGTTCCCTAAGGGTTGGGGATGACCCTTGGGTTTGTCTTTGGGTGTTACCTTGAGTGTCTCTCtg                                    <  1:157103/289‑1 (MQ=255)`  
 `tacgaaTAAAGAGCCTCTAATAGTCTATCCTAAGGTCTATACCTAAAGATAGGCCATCCTATCA‑GTGTCCCCGAAAGAGGGTCTTAGAGACGGCCTATGGAGTTCCTATAGGGTCCTTTAAAATATACCATAAAAATCTGAGTGACTATCGCACAGTGTACGGACCTAAAGTTCCCCAATAGGGGGAACCTAAAGCACAGCCAATCACCTAAAGTCAACCTTCGGATGACCTTGAGGGTTCCCTAAGGGTTGGGGATGACCCTTGGGTTTGTCTTAGGGTGTTACCTTGAGTGTCTCTCtg                                    <  1:569665/296‑1 (MQ=25)`  
 `taccaataaaagtcctacaaTGGTCCATCCTAAGGTCTATACCTAAAGATAGGCCATCCTATCA‑GTGTCACCTAAAGATGGTCTTAGAGAGGGCCTATGGAGTTCCTATAGGGTCCTTTAAAATATACCATAAAAATCTGAGTGACTATCTCACAGTGTACGGACCTAAAGTTCCCTCATAGGGGGTACCTAAAGCCCAGCCAATCACCGAAAGTCAACCTTCGGTTGACCTTGAGGGTTCCCTAAGGGTTGGGGATGACCCTTGGGTTTGTCTTTGGGTGTTACCTTGAGTGTCTCTCtg                                    <  1:123924/283‑1 (MQ=255)`  
 `taagagacaggagCCTCTAATGGTCTATCCTAAGGTCTATACCTAAAGATAGGCCATCCTATCA‑GTGTCACCTAAAGAGGGTCTTAGAGAGGGCCTATGGAGTTCCTATAGGGTCCTTTAAAATATACCATAAAAATCTGAGTGACTATCTCACAGTGTACGGACCTAAAGTTCCCCCATAGGGGGTACCTAAAGCCCAGCCAATCACCTAAAGTCAACCTTCGGTTGACCTTGAGGGTTCCCTAAGGGTTGGGGATGACCCTTAGGTTTGTCTTTGGGTGTTACCTTGAGTGTCTCTCtg                                    <  1:218826/291‑1 (MQ=255)`  
 `taaccATAAAGAGCCTCTAATGGTCTATCCTAAGGTCTATACCTAAAGATAGGCCATCCTATCA‑GTGTCACCTAAAGAGGGTCTTAGAGAGGGCCTATGGAGTTCCTATAGGGTCCTTTAAAATATACCATAAAAATCTGAGTGACTATCTCACAGTGTACGGACCTAAAGTTCCCCCATAGGGGGTACCTAAAGCCCAGCCAATCACCTAAAGTCAACCTTCGGTTGACCTTGAGGGTTCCCTAAGGGTTGGGGATGACCCTTGGGTTTGTCTTTGGGTGTTACCTTGAGTGTCTCTCtg                                    <  1:491009/298‑1 (MQ=255)`  
 `taCCCATCTAGAGCCTCTAATTGTTTATTCTAAGGTCTATCCCTACAGATAGGCCATCCTATCA‑GTGTCACCTACAGAGGGTCTTAGAGAGGGCCTATGGAGTTCCTATAGGGTCCTTTAAAATATACCATAAAAATCTGAGTGACTATCTCACAGTGTACGGACCTAAAGTTCCCCCATAGGGGGTACCTAAAGCCCAGCCAATCACCTAAAGTCAACCTTCGGTTGACCTTGAGGGTTCCCTAAGGGTTGGTGATGACCCTTGGGTTTGTCTTTGGGTGTTACCTTGAGTGTCTCTCtg                                    <  1:183432/301‑1 (MQ=255)`  
 `taCCCATAAATCTCCTCTTATGTTCGATCCCAAGGTCTATACCTAAAGAGAGGCCATCCTTTCA‑GTGTCACCTAATGGGTGTCTTAGAGACGGCCTATGGAGTTCCTATAGGGTCCTTTAAAATGTACCATAAAAATCTGAGTGACTATCTCACAGTTTACGGACCTAAAGTTCCGCCATAGGGGGTACCTAAAGCCCAGCCAATCACCTAAAGTCAACCTTCGGTTGACCTTGAGGGTTCCCTAAGGGTTGGGGATGACCCTTGGGTTTGTCTTTGGGTGTTACCTTGAGTGTCTCTCtg                                    <  1:125133/301‑1 (MQ=21)`  
 `taCCCATAAAGGGCCACCAATTGTTTATTCCAATGTTTAAACCTAAAGAAAGGGCATCCCTTCACGTGTC‑CCTAAAGTTGGGTTTAAGGAGGGCCGATGGAGGTTCTATAGGGTTCTTTAAAATTTACCAACAAAATCCGAGTTACTATCTCCCAGTTGACGGACCCTAAGTTCCCCCATAGGGGGTACCTAAAGCCCAGCCAATCACCTAAAGTCAACCTTCGGTTGACCTTGAGGGTTCCCTAAGGGTTGGGGATGACCCTTGGGTTTGTCTTTGGGTGTTACCTTGAGTGTCTCTCtg                                    <  1:670050/301‑1 (MQ=17)`  
 `taCCCATAAAGCGCCTCTAATGGTCTATCCTAAGGTCTATACCTAAAGATAGGCCATCCTATCA‑GTGTCACCTAAAGAGGGTCGTAGAGAGGGCCTATGGAGTTCCTATAGGGTCCTTTAAAATATACCATAAAAATCTGAGTGACTATCTCACAGTGTACGGACCTAAAGTTCCCCCATAGGGGGTACCTAAAGCCCAGCCAATCACCTAAAGTCAACCTTCGGTTGACCTTGAGGGTTCCCTAAGGGTTGGGGATGACCCTTGGGTTTGTCTTTGGGTGTTACCTTGAGTGTCTCTCtg                                    <  1:260148/301‑1 (MQ=255)`  
 `taCCCATAAAGAGCGTCTAATGGATTATCCTAAGGTCTATACCTAAAGATATGCCATCCTATCA‑GTGTCACCTAAAGAGGGTCTGAGAGAGGGCCTATGGAGTTACTATAGGGTCCTTTAAAATATAACATAAAAATATGAGTGACTATCTCACAGTTGACGGACCTAAAGTTCCCCCATAGGGGGTACCTAAAGCCCAGCCAATCACCTAAAGTCAACCTTCGGTTGACCTTGAGGGTTCCCTAAGGGTTGGGGATGACCCTTGGGTTTGTCTTTGGGTGTTACCTTGAGTGTCTCTCtg                                    <  1:352149/301‑1 (MQ=255)`  
 `taCCCATAAAGAGCCTCTAATGGTCTATCCTAAGGTCTATACCTAAAGATAGGCCATCCTATCA‑GTGTCACCTAAAGAGGGTCTTAGAGAGGGCCTATGGAGTTCCTATAGGGTCCTTTAAAATATACCATAAAAATCTGAGTGACTATCTCACAGTGTACGGACCTAAAGTTCCCCCATAGGGGTTACCTAAAGCCCAGCCAATCACCTAAAGTCAACCTTCGGTTGACCTTGAGGGTTCCCTAAGGGTTGGGGATGACCCTTGGGTTTGTCTTTGGGTGTTACCTTGAGTGTCTCTCtg                                    <  1:8681/301‑1 (MQ=255)`  
 `taCCCATAAAGAGCCTCTAATGGTCTATCCTAAGGTCTATACCTAAAGATAGGCCATCCTATCA‑GTGTCACCTAAAGAGGGTCTTAGAGAGGGCCTATGGAGTTCCTATAGGGTCCTTTAAAATATACCATAAAAATCTGAGTGACTATCTCACAGTGTACGGACCTAAAGTTCCCCCATAGGGGGTACCTAAAGCCCAGCCAATCACCTAAAGTCAACCTTCGGTTGACCTTGATGGTTCCCTAAGGGTTGGGGATGACCCTTGGGTTTGTCTTTGGGTGTTACCTTGAGTGTCTCTCtg                                    <  1:599584/301‑1 (MQ=255)`  
 `taCCCATAAAGAGCCTCTAATGGTCTATCCTAAGGTCTATACCTAAAGATAGGCCATCCTATCA‑GTGTCACCTAAAGAGGGTCTTAGAGAGGGCCTATGGAGTTCCTATAGGGTCCTTTAAAATATACCATAAAAATCTGAGTGACTATCTCACAGTGTACGGACCTAAAGTTCCCCCATAGGGGGTACCTAAAGCCCAGCCAATCACCTAAAGTCAACCTTCGGTTGACCTTGAGGGTTCCCTAAGGGTTGGGGATGACCCTTGGGTTTGTCTTTGGGTGTTACCTTGAGTGTCTCTCtg                                    <  1:199403/301‑1 (MQ=255)`  
 `taCCCATAAAGAGCCTCTAATGGTCTATCCTAAGGTCTATACCTAAAGATAGGCCATCCTATCA‑GTGTCACCTAAAGAGGGTCTTAGAGAGGGCCTATGGAGTTCCTATAGGGTCCTTTAAAATATACCATAAAAATCTGAGTGACTATCTCACAGTGTACGGACCTAAAGTTCCCCCATAGGGGGTACCTAAAGCCCAGCCAATCACCTAAAGTCAACCTTCGGTTGACCTTGAGGGTTCCCTAAGGGTTGGGGATGACCCTTGGGTTTGTCTTTGGGTGTTACCTTGAGTGTCTCTCtg                                    <  1:281331/301‑1 (MQ=255)`  
 `taCCCATAAAGAGCCTCTAATGGTCTATCCTAAGGTCTATACCTAAAGATAGGCCATCCTATCA‑GTGTCACCTAAAGAGGGTCTTAGAGAGGGCCTATGGAGTTCCTATAGGGTCCTTTAAAATATACCATAAAAATCTGAGTGACTATCTCACAGTGTACGGACCTAAAGTTCCCCCATAGGGGGTACCTAAAGCCCAGCCAATCACCTAAAGTCAACCTTCGGTTGACCTTGAGGGTTCCCTAAGGGTTGGGGATGACCCTTGGGTTTGTCTTTGGGTGTTACCTTGAGTGTCTCTCtg                                    <  1:183442/301‑1 (MQ=255)`  
 `taCCCATAAAGAGCCTCTAATGGTCTATCCTAAGGTCTATACCTAAAGATAGGCCATCCTATCA‑GTGTCACCTAAAGAGGGTCTTAGAGAGGGCCTATGGAGTTCCTATAGGGTCCTTTAAAATATACCATAAAAATCTGAGTGACTATCTCACAGTGTACGGACCTAAAGTTCCCCCATAGGGGGTACCTAAAGCCCAGCCAATCACCTAAAGTCAACCTTCGGTTGACCTTGAGGGTTCCCTAAGGGTTGGGGATGACCCTTGGGTTTGTCTTTGGGTGTTACCTTGAGTGTCTCTCtg                                    <  1:551185/301‑1 (MQ=255)`  
 `taCCCATAAAGAGCCTCTAATGGTCTATCCTAAGGTCTATACCTAAAGATAGGCCATCCTATCA‑GTGTCACCTAAAGAGGGTCTTAGAGAGGGCCTATGGAGTTCCTATAGGGTCCTTTAAAATATACCATAAAAATCTGAGTGACTATCTCACAGTGTACGGACCTAAAGTTCCCCCATAGGGGGTACCTAAAGCCCAGCCAATCACCTAAAGTCAACCTTCGGTTGACCTTGAGGGTTCCCTAAGGGTTGGGGATGACCCTTGGGTTTGTCTTTGGGTGTTACCTTGAGTGTCTCTCtg                                    <  1:570234/301‑1 (MQ=255)`  
 `taCCCATAAAGAGCCTCTAATGGTCTATCCTAAGGTCTATACCTAAAGATAGGCCATCCTATCA‑GTGTCACCTAAAGAGGGTCTTAGAGAGGGCCTATGGAGTTCCTATAGGGTCCTTTAAAATATACCATAAAAATCTGAGTGACTATCTCACAGTGTACGGACCTAAAGTTCCCCCATAGGGGGTACCTAAAGCCCAGCCAATCACCTAAAGTCAACCTTCGGTTGACCTTGAGGGTTCCCTAAGGGTTGGGGATGACCCTTGGGTTTGTCTTTGGGTGTTACCTTGAGTGTCTCTCtg                                    <  1:486006/301‑1 (MQ=255)`  
 `taCCCATAAAGAGCCTCTAATGGTCTATCCTAAGGTCTATACCTAAAGATAGGCCATCCTATCA‑GTGTCACCTAAAGAGGGTCTTAGAGAGGGCCTATGGAGTTCCTATAGGGTCCTTTAAAATATACCATAAAAATCTGAGTGACTATCTCACAGTGTACGGACCTAAAGTTCCCCCATAGGGGGTACCTAAAGCCCAGCCAATCACCTAAAGTCAACCTTCGGTTGACCTTGAGGGTTCCCTAAGGGTTGGGGATGACCCTTGGGTTTGTCTTTGGGTGTTACCTTGAGTGTCTCTCtg                                    <  1:184836/301‑1 (MQ=255)`  
 `taCCCATAAAGAGCCTCTAATGGTCTATCCTAAGGTCTATACCTAAAGATAGGCCATCCTATCA‑GTGTCACCTAAAGAGGGTCTTAGAGAGGGCCTATGGAGTTCCTATAGGGTCCTTTAAAATATACCATAAAAATCTGAGTGACGATCTCACAGTGTACGGACCTAAAGTTCCCCCATAGGGGGTACCTAAAGCCCAGCCAATCACCTAAAGTCAACCTTCGGTTGACCTTGAGGGTTCCCTAAGGGTTGGGGATGACCCTTGGGTTTGTCTTTGGGTGTTACCTTGAGTGTCTCTCtg                                    <  1:276539/301‑1 (MQ=255)`  
 `taCCCATAAAGAGCCTCTAATGGTCTATCCTAAGGTCTATACCTAAAGATAGGCCATCCTATCA‑GTGTCACCTAAAGAGGGTCTTAGAGAGGGACTATGGAGTTCCTATAGGGTCATTTAAAATATACCATAAAAATCTGAGTGACTATCTCACAGTGTACGGAACTAAAGTTCCCCCATAGGGGGTACCTAAAGCCCAGCCAATCACCTAAAGTCAACCTTCGGTTGACCTTGAGGGTTCCCTAAGGGTTGGGGATGACCCTTGGGTTTGTCTTTGGGTGTTACCTTGAGTGTCTCTCtg                                    <  1:608394/301‑1 (MQ=255)`  
 `taCCCATAAAGAGCCTCTAATGGTCTATCCTAAGGTCTATAACTAAAGATAGGCCATCCTATCA‑GTGTCACCTAAAGAGGGTCTTAGAGAGGGCCTATGGAGTTCCTATAGGGTCCTTTAAATTATACCATAAAAATCTGAGGGACTATCTCACAGTGTACGGACCTAAAGTTCCCCCATAGGGGGTACCTAAAGCCCAGCCAATCACCTAAAGTCAACCTTCGGTTGACCTTGAGGGTTCCCTAAGGGTTGGGGATGACCCTTGGGTTTGTCTTTGGGTGTTACCTTGAGTGTCTCTCtg                                    <  1:370684/301‑1 (MQ=255)`  
 `taCCCAGAAAGAGCCTCTAATGGTCTATCCTAAGGTCTATACCTAAAGATAGGCCATCCTATCA‑GTGTCACCTAAAGAGGGTCTTAGAGGGGGCCTATGGAGTTCCTATAGGGTCCTTTAAAATATACCATAAAAATCTGTGTGACTATCTCACAGTGTACGGACCTAAAGTTCCCCCATAGGGGGTACCTAAAGCCCAGCCAATCACCTAAAGTCAACCTTCGGTTGACCTTGAGGGTTCCCTAAGGGTTGGGGATGACCCTTGGGTTTGTCTTTGGGTGTTACCTTGAGTGTCTCTCtg                                    <  1:83378/301‑1 (MQ=255)`  
 `gtataatagacagCCTCTAATGGTCTATCCTAAGGTCCATACCTAAAGATAGGCCATCCTATCA‑GTGTCACCTAAAGAGGGTCTAAGAGAGGGCCTATGGAGTTCCTATAGGGTCCTTTAAAATATACCATAAAAATCTGAGTGACTATCTCACAGTGTACGGACCTAAAGTTCCCCCATAGGGGGTACCTAAAGCCCAGCCAATCACCTAAAGTCAACCTTCGGTTGACCTTGAGGGTTCCCTAAGGGTTGGGGATGACCCTTGGGTTTGTCTTTGGGTGTTACCTTGAGTGTCTCTCtg                                    <  1:262294/290‑1 (MQ=255)`  
 `ggctcgtagatgtgtataagagacagaTCCTAAGGTCTATACCTAAAGATAGGCCATCCTATCA‑GTGTCACCTAAAGAGGGTCTTAGAGAGGGCCTATGGAGTTCCTATAGGGTCCTTTAAAATATACCATAAAAATCTGGGTGCCTAGCTCACAGTGTACGGACCTAAAGTTCCCCCATAGGGGGTACCTAAAGCCCAGCCAATCACCTAAAGTCAACCTTCGGTTGACCTTGAGGGTTCCCTAAGGGTTGGGGATGACCCTTGGGTTTGTCTTTGGGTGTTACCTTTAGTGTCTCTCtg                                    <  1:463949/275‑1 (MQ=21)`  
 `ggctcggagatgtgtataagagacagaTCCTAAGGTCTATACCTAAAGATAGGCCATCCTATCA‑GTGTCACCTAAAGAGGGTCTTAGAGAGGTCCTATGGAGTTCCTATAGGGTCCTTTAAAATATACCATAAAAATCTGAGTGACTATCTCACAGTGTACGGACCTAAAGTTCCCCCATAGGGGGTACCTAAAGCCCAGCCAATCACCTAAAGTCAACCTTCGGTTGACCTTGAGGGTTCCCTAAGGGTTGGGGATGACCCTTGGGTTTGTCTTTGGGTGTTACCTTGAGTGTCTCTCtg                                    <  1:478682/275‑1 (MQ=255)`  
 `ggctcggagatgtgtataagagacagaTCCTAAGGTCTATACCTAAAGATAGGCCATCCTATCA‑GTGTCACCTAAAGAGGGTCTTAGAGAGGGCCTATGGAGTTCCTATAGGGTCCTTTAAAATATACCATAAAAATCTGAGTGACTATCTCACAGTGTACGGACCTAAAGTTCCCCCATAGGGGGTACCTAAAGCCCAGCCAATCACCTAAAGTCAACCTTCGGTTGACCTTGAGGGTTCCCTAAGGGTTGGGGATGACCCTTGGGTTTGTCTTTGGGTGTTACCTTGAGTGTCTCTCtg                                    <  1:215725/275‑1 (MQ=255)`  
 `ggctcggagaagagtataagagacggaTCCTAAGGTCTATACCTAAAGATAGGCCATCCTATCA‑GTGTCACCTAAAGAGGGTCTTAGAGAGGGCCTATGGAGTTCCTATAGGGTCCTTTAAAATATACCATAAAAATCTGAGTGACTATCTCACAGTGTACGGACCTAAAGTTCCCCCATAGGGGGTACCTAAAGCCCAGCCGATCACCTAAAGTCAACCTTCGGTTGACCTTGAGGGTTCCCTAAGGGTTGGGGATGACCCTTGGGTTTGTCTTTGGGTGTTACCTTGAGTGTCTCTCtg                                    <  1:310121/275‑1 (MQ=255)`  
 `ggagatgtgtataagagacagggTATATCCTAAGGTCTATACATAAAGATAGGCCATCCTATCA‑GTGTCACCTAAAGAGGGTCTTAGAGAGGGCCTATGGAGTTCCTATAGGGTCCTTTAAAATATACCATAAAAATCTGAGGGACTATCTCACAGTGTACGGACCTAAAGTTCCCCCATAGGGGGTACCTAAAGCCCAGCCAATCACCTAAAGTCAACCTTCGGTTGACCTTGAGGGTTCCCTAATGGTTGGGGATGACCCTTGGGTTTGTCTTTGGGTGTTACCTTGAGTGTCTCTCtg                                    <  1:437006/280‑1 (MQ=25)`  
 `ggaaatgtgtataagagacagggTCTATCCTAAGGTATATACCTAAAGATAGTCCATCATATCC‑GTGGCACCTAAAGAGGGTCTTAGAGAGGGGCTATGGAGTTCCTATAGGGTCCTTTAAAATATACCATAAAAATCTTAGTGACGATCTCACAGTGTACGGACCTAAAGTTCCCCCATAGGGGGTACCTAAAGCCCAGCCAATCACCTAAAGTCAACCTTCGGTTGACCTTGAGGGTTCCCTAAGGGTTGGGGATGACCCTTGGGTTTGTCTTTGGGTGTTACCTTGAGTGTCTCTCtg                                    <  1:67779/280‑1 (MQ=21)`  
 `gactcggatatgtgtataagatgcagaTCCTAAGATCTATACTGAAAGATAGGCCATCCTATCA‑GTGTCACCTAAAGAGGGTCTTAGAGAGGGCCTATGGAGTTCCTATAGGGTCCTTTAAAATATACCATAAAAATCTGAGTGACTATCTCACAGTGTACGGACCTAAAGTTCCCCCATAGGGGGTACCTAAAGCCCAGCCAATCACCTAAAGTCAACCTTCGGTTGACCTTGAGGGTTCCCTAAGGGTTGGGGATGACCCTTGGGTTTGTCTTTGGGTGTTACCTTGAGTGTCTATCtg                                    <  1:462677/275‑1 (MQ=21)`  
 `cggagatgtgtataagagacagttCTATCCGAAGGTCTATACCTAAAGATAGGCCATCCTATCA‑GTGTCACCTAAAGAGGGTCTTAGAGAGGGACTATGGAGTTCCTATAGGGTCCTTTAAAATATACCATAAAAATCTGAGTGACTATCTCACAGTGTACGGACCTAAAGTTCCCCCATAGGGGGTACCTAAAGCCCAGCCAATCACCTAAAATCAACCTTCGGTTGACCTTGAGGGTTCCCTAAGGGTTGGGGATGACCCTTGGGTTTGTCTTTGGGTGTTACCTTGAGTGTCTCTCtg                                    <  1:144625/278‑1 (MQ=25)`  
 `cggagatgtgtataagagacaggTCTATCCGAAGGTCTATACCTAAAGATAGGCCATCCTATCA‑GTGTCACCTAAAGAGGGTCTTAGAGAGGGCCTATGGAGTTCCTATAGGGTCCTTTAAAATATACCATAAAAATCTGAGTGACTATCTCACAGTGTACGGACCTAAAGTTCCCCCATAGGGGGTACCTAAAGCCCAGCCAATCACCTAAAGTCAACCTTCGGTTGACCTTGAGGGTTCCCTAAGGGTTGGGGATGACCCTTGGGTTTGTCTTTGGGTGTTACCTTGAGTGTCTCTCtg                                    <  1:366539/280‑1 (MQ=255)`  
 `cgcccATTAAGCGCCTCTATTGGTCTATCCTAATGTCTATACCTAAAGATAGGCCCTCCTATCC‑GGGTTACCTAAAGTGGGTCTTAGGGAGGGCCTATGGAGTTCCTATAGGGTCCTTTAAAATATACCATAAAAATCTGAGTGACTATCTCACAGTGTACGGACCTAAAGTTCCCCCATAGGGGGTACCTAAAGCCCAGCCAATCACCTAAAGTCAACCTTCGGTTGACCTTGAGGGTTCCCTAAGGGTTGGGGATGACCCTTGGGTTTGTCTTTGGGTGTTACCTTGAGTGTCTCTCtg                                    <  1:145374/299‑1 (MQ=21)`  
 `cgcccATAAAGAGCCTCTAATGGTCTATCCTAAGGTCTATACCTAAAGATAGGCCATCCTATCA‑GTGTCACCGAAAGAGGGTCTTAGAGAGGGCCTATGGAGTTCCTATAGGGTCCTTTAAAATATACCATAAAAATCTGAGTGACTATCTCACAGTGTACTGACCTAAAGTTCCCCCATAGGGGGTACCTAAAGCCCAGCCAATCACCTAAAGTCAACCTTCGGTTGACCTTGAGGGTTCCCTAAGGGTTGTGGATGACCCTTGGGTTTGTCTTTGGGTGTTACCTTGAGTGTCTCTCtg                                    <  1:427853/299‑1 (MQ=255)`  
 `atccactAAAGTGCCTCTAATGGTCTATCCTAAGTCCGATACCTAAAGATAGGCCATCCTATCA‑GTGTCACCTAAAGAGGGTCTTAGAGATGGCCTATGGAGTTCCTATAGTGTCCTTTAAAATATACCATAAAAATCTGAGTGACTATCTCACAGTGTACGGACCTAAAGTTCCCCCATAGGGGGTACCTAAAGCCCAGCCAATCACCTAAAGTCAACCTTCGGTTGACCTTGAGGGTTCCCTAAGGGTTGGGGATGACCCTTGGGTTTGTCTTTGGGTGTTACCTTGAGTGTCTCTCtg                                    <  1:630968/295‑1 (MQ=255)`  
 `agctcATAAAGAGCCTCTAATGGTCTATCCTAAGGTCTATACCTAAAGATAGGCCATCCTATCA‑GTGTCACCTAAAGAGGGTCTTAGAGAGGGCCTATGGAGTTCCTATAGGGTTCTTTAAAATATACCATAAAAATCTGAGTGACTATCTCACAGTGTACGGACCTAAAGTTCCCCCATAGGGGGTACCTAAAGCCCAGCCAATCACCTAAAGTCAACCTTCGGTTGGCCTTGAGGGTTCCCTAAGGGTTGGGGATGACCCTTGGGTTTGTCTTTGGGTGTTACCTTGAGTGTCTCTCtg                                    <  1:144112/297‑1 (MQ=255)`  
 `agcccATTAAGAGCCTCTAATAGTCTATCCTAAGGTCTATACCTAAAGATAGGCCATCCTATCA‑GTGTCACCTAAAGAGGGTCTTAGAGAGGGCCTATGGAGTTCCTATAGGGTCCTTTAAAATATACCATAAAAATCTGAGTGACTATCTCACAGTGTACGGACCTAAAGTTCCCCCATAGGGGGTACCTAAAGCCCAGCCAATCACCTAAAGTCAACCTTCGGTTGACCTTGAGGGTTCCCTAAGGGTTGGGGATGACCCTTGGGTTTGTCTTTGGGTGTTACCTTGAGTGTCTCTCtg                                    <  1:403697/299‑1 (MQ=255)`  
 `agcccATAAAGAGCCTCTAATGGTCTATCCTAAGGTCTATACCTAAAGATAGGCCATCCTATCA‑GTGTCACCTAAAGAGGGTCTTAGAGAGGGCCTATGGAGTTCCTATAGGGTCCTTTAAAATATACCATAAAAATCTGAGTGACTATCTCACAGTGTACGGACCTAAAGTTCCCCCATAGGGGGTACCTAAAGCCCAGCCAATCACCTAAAGTCAACCTTCGGTTGACCTTGAGGGTTCCCTAAGGGTTGGGGATGACCCTTGGGTTTGTCTTTGGGTGTTACCTTGAGTGTCTCTCtg                                    <  1:120100/299‑1 (MQ=255)`  
 `agcccATAAAGAGCCTCTAATGGTCTATCCTAAGGTCTATACCTAAAGATAGGCCATCCTATCA‑GTGTCACCTAAAGAGGGTCTTAGAGAGGGCCTATGGAGTTCCTATAGGGTCCTTTAAAATATACCATAAAAATCTGAGTGACTATCTCACAGTGTACGGACCTAAAGTTCCCCCATAGGGGGTACCTAAAGCCCAGCCAATCACCTAAAGTCAACCTTCGGTTGACCTTGAGGGTTCCCTAAGGGTTGGGGATGACCCTTGGGTTTGTCTTTGGGTGTTACCTTGAGTGTCACTCtg                                    <  1:612751/299‑1 (MQ=255)`  
 `agcccATAAAGAGCCTCTAATGGTCTATCCTAAGGTCTATACCTAAAGATAGGCCATCCTATCA‑GTGTCACCTAAAGAGGGTCTTAGAGAGGGCCTATGGAGTTCCTATAGGGTCCTTTAAAATATACCATAAAAATCTGAGTGACTATCTCACAGTGTACGGACCTAAAGTTCCCCCACAGGGGGTACCTAAAGCCCAGCCAATCACCTAAAGTCAACCTTCGGTTGACCTTGAGGGTTCCCTAAGGGTTGGGGATGACCCTTGGGTTTGTCTTTGGGTGTTACCTTGAGTGTCTCTCtg                                    <  1:13783/299‑1 (MQ=255)`  
 `agcccATAAAGAGCCTCTAATGGTCTATCCTAAGGTCTATACCTAAAGATAGGCCATCCTATCA‑GTGTCAACTAAAGAGGGTCTTAGAGAGGGCCTATGGAGTTCCTATAGGGTCCTTTGAAATATACCATAAAAATCTGAGTGACTAACTCACAGTGTACGGACCTAAAGTTCCCCCATAGGGGGTACCTAAAGCCCAGCCAATCACCTAAAGTCAACCTTCGGTTGACCTTGAGGGTTCCCTAAGGGTTGGGGATGACCCTTGGGTTTGTCTTTGGGTGTTACCTTGAGTGTCTCGCtg                                    <  1:498229/299‑1 (MQ=255)`  
 `agcccATAAAGAGCCTATAATGGTCTATCCTAAGGTCTATACCTAAAGATAGGCCATCCTATCA‑GTGTCACCTAAAGAGGGTCTTAGAGAGGGCCTATGGAGTTCCTATAGGGTCCTTTAAAATATACCATAAAAATCTGAGTGACTATCGCACAGTGTACGGACCTAAAGTTCCCCCATAGGGGGTACCTAAAGCCCAGCCAATCACCTAAAGTAAACCTTCGGTTGACCTGGAGGGTTCCCTAAGGGTTGGGGATGACCCTTGGGGTTGTCTTTGGGTGTTACCTTGAGTGTCTCTCtg                                    <  1:223969/299‑1 (MQ=255)`  
 `agcccATAAAGAACCTCTAATGGTCTATCCTAAGGTCTATACCTAAAGATAGGCCATCCTATCA‑GTGTCACCTAAAGTGGGTCTTAGAGAGGGCCTATGGAGTTCCTATATGGTCCTTTAAAATATACCATAAATATCTGAGTGACTATCTCACAGTGTACGGACCTAAAGTTCCCCCATAGGGGGTACCTAAAGCCCAGCCAAGCACCTAAAGTCAACCTTCGGTTGACCTTGAGGGTTCCCTAAGGGTTGGGGATGACCCTTGGGTTTGTCTTTGGGTGTTACCTTGAGTGTCTCTCtg                                    <  1:416239/299‑1 (MQ=255)`  
 `aaccactAAAGGGCCTCTAATGGTCTATCCGAAGGTCTATACCTAAAGATAGGCCATCCTGTCA‑GTGTCACCTAAAGAGGGTCTTAGAGAGGGCCTATGGAGTTCCTATAGGGTCCTTTAAAATATACCATAAAAATCTGAGTGACTATCTCACAGTGTACGGACCTAAAGTTCCCCCATAGGGGGTACCTAAATCCCAGCCAATCACCTAACGTCAACCTTCGGTTGACCTTGAGTGTTCCCTAAGGGTTGGGGATGACCCTTGGGTTTGTCTTTGGGTGTTACCTTGAGTGTCTCTCtg                                    <  1:87483/295‑1 (MQ=255)`  
 `aaaccATAAAGAGCCTCTAAGGGTCTATCCTAAGGTCTATACCTAAAGATAGGCCATACTATGA‑GTGTCACCGAAAGAGGGTCTTAGAGAGGGCCTATGGAGTTCCTATAGGGTCCTTTAAAATATACCATAAAAATCTGAGTGACTATCTCACAGTGTACGGACCTAAAGTTCCCCCATAGGGGGTACCTAAAGCCCAGCCAATCACCTAAAGTAAACCTTCGGTTGACCTTGAGGGTTCCCTAAGGGTTGGGGATGACCCTTGGGTTTGTCTGTGGGTGTTACCTTGAGTGTCTCTCtg                                    <  1:182217/298‑1 (MQ=255)`  
 `aaCCCATTAAGAGCCTCTAATGGTCTATCCTAAGGGATATACCTAAAGATAGGCCATCCTATCA‑GTGTCACCTAAAGAGTGTCTTAGAGAGGGCCTATGGAGTTCCTATAGGGTTCTTTAAAATATACCATCAAAATCTGAGTGACTATCTCACAGTGTACGGACCTAAAGTTCCCCCATAGGGGGTACCTAAAGCCCAGCCAATCACCTAAAGTCAACCTTCGGTTGACCTTTAGGGTTCCCTAAGGGTTGGGGATGACCCTTGGGTTTGTCTTTGGGTGTTACCTTGAGTGTCTCTCtg                                    <  1:667724/300‑1 (MQ=255)`  
 `aaCCCATAACGAGCCTCTAATGGTCTATCCTAAGGTCTATACCTAAAGATAGGCCATCCTATCA‑GTGTCACCTAAAGAGGGTCTTAGAGAGTGCCTATGGAGTTCCTATAGGGTCCTTTAAAATATACCATAAGAATCTGGGTGACTATCTCACAGTGTACGGACCTAAAGTTCCCCCATAGGGGGTACCTAAAGCCCAGCCAATCACCTAAAGTCAACCTTCGGTTGACCTTGAGGGTTCCCTAAGGGTTGGGGATGACCCTTGGGTTTGTCTTTGGGTGTTACCTTGAGTGTCTCTCtg                                    <  1:168752/300‑1 (MQ=255)`  
 `aaCCCATAAAGGGCCTCTAATGGTCTATCCTAAGGTCTATACCTAAAGATAGGCCATCCTATCA‑GTGTCACCTAAAGAGGGTCTTAGAGAGGGCCTATGGAGTTCCTATAGGGTCCTTTAAAATATACCATAAAAATCTGAGTGACTATCTCACAGTGTACGGACCTAAAGTTCCCCCATAGGGGGTACCTAAAGCCCAGCCAATCACCTAAAGTCAACCTTCGGTTGACCTTGAGGGTTCCCTAAGGGTTGGGGATGACCCTTGGGTTTGTCTTTGGGTGTTACCTTGAGTGTCTCTCtg                                    <  1:426606/300‑1 (MQ=255)`  
 `aaCCCATAAAGAGCCTCTAATTGTCAATCCTAAGGGCTATACCTAAAGATAGGCCATCCTATCA‑GTGGCACCTAAAGAGGGTCTTAGAGACGGCCTATGGAGTTCCTATAGGGTCCTTTAAAATATATCATAAAAATCTGAGTGACTATCTCACAGTGTACGGACCTAAAGTTCCCCCATAGGGGGTACCTAAAGCCCAGCCAATCACCTAAAGTCAACCTTCGGTTGACCTTGAGGGTTCCCTAAGGGTTGGGGATGACCCTTGGGTTTGTCTTTGGGTGTTACCTTGAGTGTCTCTCtg                                    <  1:220110/300‑1 (MQ=255)`  
 `aaCCCATAAAGAGCCTCTAATGGTCTATCCTAAGGTCTATACCTAAAGATAGGCCATCCTATCA‑GTTTCACCTAAAGAGGGTCTTAGAGAGGGCCTATGGAGTTCCTATAGGGTCCTTTAAAATATACCATAAAAATCTGAGTGACTATCTCACAGTGTACGGACCTAAAGTTCCCCCATAGGGGGTACCTAAAGCCCAGCCAATCACCTAAAGTCAACCTTCGGTTGACCTTGAGGGTTACCTAAGGGTTGGGGATGACCCTTGGGTTTGTCTTTGGGTGTTACCTTGAGTGTCTCTCtg                                    <  1:518607/300‑1 (MQ=255)`  
 `aaCCCATAAAGAGCCTCTAATGGTCTATCCTAAGGTCTATACCTAAAGATAGGCCATCCTATCA‑GTGTCACCTAAAGAGGGTCTTAGAGAGGGCCTATGGAGTTCCTATAGGGTCCTTTAAAATATACCATAAAAATCTGAGTGACTATCTCACAGTGTACGGACCTAAAGTTCCCCCATAGGGGGTACCTAAAGCCCAGCCAATCACCTAAAGTCAACCTTCGGTTGTCCTTGAGGGTTCCCTAAGGGTTGGGGATGACCCTTGGGTTTGTCTTTGGGTGTTACCTTGAGTGTCTCTCtg                                    <  1:551522/300‑1 (MQ=255)`  
 `aaCCCATAAAGAGCCTCTAATGGTCTATCCTAAGGTCTATACCTAAAGATAGGCCATCCTATCA‑GTGTCACCTAAAGAGGGTCTTAGAGAGGGCCTATGGAGTTCCTATAGGGTCCTTTAAAATATACCATAAAAATCTGAGTGACTATCTCACAGTGTACGGACCTAAAGTTCCCCCATAGGGGGTACCTAAAGCCCAGCCAATCACCTAAAGTCAACCTTCGGTTGACCTTGAGGGTTCCCTAAGGGTTGGGGATGACCCTTGGGTTTGTCTTTGGGTGTTACCTTGAGTGTCTCTCtg                                    <  1:118091/300‑1 (MQ=255)`  
 `aaCCCATAAAGAGCCTCTAATGGTCTATCCTAAGGTCTATACCTAAAGATAGGCCATCCTATCA‑GTGTCACCTAAAGAGGGTCTTAGAGAGGGCCTATGGAGTTCCTATAGGGTCCTTTAAAATATACCATAAAAATCTGAGTGACTATCTCACAGTGTACGGACCTAAAGTTCCCCCATAGGGGGTACCTAAAGCCCAGCCAATCACCTAAAGTCAACCTTCGGTTGACCTTGAGGGTTCCCTAAGGGTTGGGGATGACCCTTGGGTTTGTCTTTGGGTGTTACCTTGAGTGTCTCTCtg                                    <  1:27251/300‑1 (MQ=255)`  
 `aaCCCATAAAGAGCCTCTAATGGTCTATCCTAAGGTCTATACCTAAAGATAGGCCATCCTATCA‑GTGTCACCTAAAGAGGGTCTTAGAGAGGGCCTATGGAGTTCCTATAGGGTCCTTTAAAATATACCATAAAAATCTGAGTGACTATCTCACAGTGTACGGACCTAAAGTTCCCCCATAGGGGGTACCTAAAGCCCAGCCAATCACCTAAAGTCAACCTTCGGTTGACCTTGAGGGTTCCCTAAGGGTTGGGGATGACCCTTGGGTTTGTCTTTGGGTGTTACCTTGAGTGTCTCTCtg                                    <  1:257984/300‑1 (MQ=255)`  
 `aaCCCATAAAGAGCCTCTAATGGTCTATCCTAAGGTCTATACCTAAAGATAGGCCATCCTATCA‑GTGTCACCTAAAGAGGGTCGTAGAGAGGGCCTATGGAGTTCCTATAGGGTCCTTTAAAATATACCATAAAAATCTGAGTGACTATCTCACAGTGTACGGACCTAAAGTTCCCCCATAGGGGGTACCTAAAGCCCAGCCAATCACCTAAAGTCAACCTTCGGTTGACCTTGAGGGTTCCCTAAGGGTTGGGGATGACCCTTGGGTTTGTCTTTGGGTGTTACCTTGAGTGTCTCTCtg                                    <  1:468286/300‑1 (MQ=255)`  
 `aaCCCATAAAGAGCCTCTAATGGTCTATCCTAAGGTCTATACCTAAAGATAGGCCATCCTATCA‑GTGTCACCTAAAGAAGGTCTTAGAGAGGGTCTATGGAGTTCCTATAGGGTCCTTTAAAATATACCATAAAAATCTGAGTGACTATCTCACAGTGTACGGACCTAAAGTTCCCCCATAGGGGGTACCTAAAGCCCAGCCAATCACCTAAAGTCAACCTTCGGTTGACCTTGAGGATTCCCGAAGGGTTGGGGATGACCCTTGGGTTTGTCTTTGGGTGTTACCTTGAGTGTCTCTCtg                                    <  1:442017/300‑1 (MQ=255)`  
 `aaCCCATAAAGAGCCTCTAATGGTCTATCCTAAGGTCTATACCTAAAGATAGACCATCCTATCA‑GTGTCACCTAAAGAGGGTCTTAGAGAGGGCCTATGGAGTTCCTATAGGGTCCTTTAAAATATACCATAAAAATCTGAGTGACTATCTCACAGTGTACGGACCTAAAGTTCCCCCATAGGGGGTACCTAAAGCCCAGCCAATCACCTAAAGTCAACCTTCGGTTGACCTTGAGGGTTCCCTAAGGGTTGGGGATGACCCTTGGGTTTGTCTTTGGGTGTTACCTTGAGTGTCTCTCtg                                    <  1:271803/300‑1 (MQ=255)`  
 `aaCCCATAAAGAGCCTCTAATGGTCTATCATAAGGTCGATACATAAAGATAGGCCATCGTATCA‑GTGGCACATAAAGAGGGTCTTAGATAGGGCCTATGGAGTTCCGATAGGGGCCTTTAAAATATACCATAAAAATCTGAGTGACTATCTCACAGTGTACGGACCTAAAGTTACCCCATAGGGGTTACCTAAAGCCCAGCCAATCACCTAAAGTCAACCTTAGGTTGACCTTGAGGGTTCCCTAAGGGTGGGGGATGACCCGTGGGTTTGTCTTTGGGTGTTACCTTGAGTGTCTCTCtg                                    <  1:392474/300‑1 (MQ=25)`  
 `aaCCCATAAAGAGCCTCTAATGGGCTATCCTAAGGTCTATACCTAAAGATAGGCCATCCTATCA‑GTGTCACCTAAAGAGGGTCTTAGAGAGGGCCTATGGAGTTCCTATAGGGTCCTTTAAAATATACCATAAAAATCTGAGTGACTATCTCACAGTGTACGGACCTAAAGTTCCCCCATAGGGGGTACCTAAAGCCCAGCCAATCACCTAAAGTCAACCTTCGGTTGACCTTGAGGGTTCCCTAAGGGTTGGGGATGACCCTTGGGTTTGTCTTTGGGTGTTACCTTGAGTGTCTCTCtg                                    <  1:186938/300‑1 (MQ=255)`  
   `tgtataagagacagctAATGGTCTATCCTAATGTCTATACCTAAAGATAGGCCATCCTATCA‑GTGTCACCTAAAGAGGGTCTTAGAGAGGGCCTATGGAGTTCCGATAGGGTCCTGTAAAATATACCATAAAAATCTGAGTGACTATCTCACAGTGTACGGACCTAAAGTTCCCCCATAGGGGGTACCTAAAGCCCAGCCAATCACCTAAAGTCAACCTTCGGTTGACCTTGAGGGTTCCCTAAGGGTTGGGGATGACCCTTGGGTTTGTCTTTGGGTGTTACCTTGAGTGTCTCTCtg‑tg                                 <  1:42958/287‑1 (MQ=255)`  
   `cccATAACGAGCCTCTAATGGTCTATCCTAAGGTCTATACCTAAAGATAGGCCATCCTATCA‑GTGTCACCTAAAGAGGGTCTTAGAGAGGGCCTATGGAGTTCCTATAGGGTCCTTTAAAATATACCATAAAAATCTGAGTGACTATCTCACAGTGTACGGACCTAAAGTTCCCCCATAGGGGGTACCTAAAGCCCAGCCAATCACCTAAAGTCAACCTTCGGTTGACCTTGAGGGTTCCCTAAGGGTTGGGGATGACCCTTGGGTTTGTCTTTGGGTGTTACCTTGAGTGTCTCTCtgct                                  >  1:126094/1‑299 (MQ=255)`  
   `cccATAAAGAGCCTCTAATGGTCTATCCTAAGGTCTATACCTAAAGATAGGCCATCCTATCA‑GTGTTACCTAAAGAGGGTCTTAGAGAGGGCCTATGGAGTTCCTATAGGGTCCTTTAAAATATACCATAAAAATCTGAGTGACTATCTCACAGTGTACGGACCTAAAGTTCCCCCATAGGGGGTACCTAAAGCCCAGCCAATCACCTAAAGTCAACCTTCGGTTGACCTTGAGGGTTTCCTAAGGGTTGGGGATGACCCTTGGGTTTGTCTTTGGGTGTTACCTTTATTGTCTCTCtgct                                  >  1:436972/1‑299 (MQ=255)`  
   `cccATAAAGAGCCTCTAATGGTCTATCCTAAGGTCTATACCTAAAGATAGGCCATCCTATCA‑GTGTCACCTAAAGAGGGTCTTAGAGAGGGCCTATGGAGTTCCTATAGGGTCCTTTAAAATATACCATAAAAATCTGAGTGACTATCTCACAGTGTACGGACCTAAAGTTCCCCCATAGGGGGTACCTAAAGCCCAGCCAATCACCTAAAGTCAACCTTCGGTTGACCTTGAGGGTTCCCTAAGGGTTGGGGATGACCCTTGGGTTTGTCTTTGGGTGTTACCTTGAGTGTCTCTCtgct                                  >  1:432509/1‑299 (MQ=255)`  
   `cccATAAAGAGCCTCTAATGGTCTATCCTAAGGTCTATACCTAAAGATAGGCCATCCTATCA‑GTGTCACCTAAAGAGGGTCTTAGAGAGGGCCTATGGAGTTCCTATAGGGTCCTTTAAAATATACCATAAAAATCTGAGTGACTATCTCACAGTGTACGGACCTAAAGTTCCCCCATAGGGGGTACCTAAAGCCCAGCCAATCACCTAAAGTCAACCTTCGGTTGACCTAGAGGGTTCCCTAAGGGTTGGGGATGACCCTTGGGTTTGTCTTTGGGTGTTACCTTGAGTGTCTCTCtgct                                  >  1:159082/1‑299 (MQ=255)`  
   `cccATAAAGAGCCTCTAATGGTCTATCCTAAGGTCTATACCTAAAGATAGGCCATCCTATCA‑GTGTCACCTAAAGAGGGTCTTAGAGAGGGCCTATGGAGTTCCTATAGGGTCCTTTAAAATATACAATAACAATCTGAGTGACTATCTCACAGTGTACGGACCTAAAGTTCCCCCATAGGGGGTACCTAAAGCCCAGCCAATCACCTAAAGTCAACCTTCGGTTGACCTTGAGGGTTCCCTAAGGGTTTGGGATGACCCTTGAGTTTTTCTTTGGGTGTTACCTTGATTGTCTCTCtg‑tt                                 >  1:370377/1‑300 (MQ=255)`  
   `cccATAAAGAGCCTCTAATGGTCTATACTAAGGTCTATACCTAAAGATAGGCCATCCTATCA‑GTGTCACCTAAAGAGGGTCTTAGAGAGGACCTATGGAGTTCCTAGAGGGTCCTTTAAAATATACCATAAAAATCTGAGTGACTATCTCACAGTGTACGGACCTAAAGTTCCCCCATAGGGGGTACCTAAAGCCCAGCCAATCACCTAAAGTCAACCTTCGGTTGACCTTGAGGGTTCCCTAAGGGTTGGGGATGACCCTTGGGTTTGTCTTTGGGTGTTACCTTGAGTGTCTCTCtg‑tg                                 <  1:593418/301‑1 (MQ=255)`  
      `aTAAAGAGCCTCTAATGGTCTATCCTAAGGTCTATACCTAAAGATAGGCCATCCTATTA‑GTGTCACCTAAAGAGGGTCTTAGAGAGGGCCTATGGAGTTCCTATAGGGTCCTTTAAAATATACCATAAAAATCTGAGTGACTATCTCACAGTGTACGGACCTAAAGTTCCCCCATATGGGGTACCTAAAGCCCAGCCAATCACCTAAAGTCAACCTTCGGTTGACCTGGAGGGTTCCCTAAGGGTTGGGTATGACCCTTGGGTTTGTCTTTGGGTGTTACCTTGAGTGTCTCTCtg‑tctgt                              >  1:625972/1‑297 (MQ=255)`  
        `aaaGAGCCTCTAATGGTCTATCCTAAGGTCTATACCTAAAGATAGGCCATCCTATCA‑GTGTCACCTAAAGAGGGTCTTAGAGAGGGCCTATGGCGTTCCTATAGGGTCCTTTAAAATATACCATAAAACTCTGAGTGACTATCTCACAGTGTACGGAACTAAAGTTCCACCATCGGGGGTACCTAAAGCCCAGCCAATCACCTAAAATCAACCTTCGGTTGACCTTGAGGGTTCCCTAAGGGTTTGGGATGACCTTTACGTTTGTCTTGGGGTGTTACCTTGAGTGTCCCTCtgctggctc                             >  1:302000/1‑294 (MQ=25)`  
           `tgtgtataagagacagaTCCTAAGGTCTATCCCTAAAGATAGGCCATCCTATCA‑GTGTCACCTAAAGAGGGTCTTAGAGAGGGCCTATGGAGTTCCTATAGGGTCCTTTAAAATATACCATAAAAATCTGAGTGACTATCTCACAGTGTACGGACCTAAAGTTCCCCCATAGGGGGTACCTAAAGCCCAGCCAATCACCTAAAGTCAACCTTCGGTTGACCTTGAGGGTTCCCTAAGGGTTGGGGATGACCCTTGGGTTTGTCTTTGGGTGTTACCTTGAGTGTCTCTCTG‑TGTCCCtatc                         <  1:12901/285‑4 (MQ=255)`  
           `gagCCTCTAATGGTCTATCCTAAGGTCTACACCTAAAGATAGGCCATCCTATCT‑GTGTCACCTAAAGAGGGTCTTAGAGAGGGCCTATGGAGTTCCTATAGGGTCCTTTAAAATATACCATAAAAATCTGAGTGACTATCTCACAGTGTACGGACCTAAAGTTCCCCCATAGGGGGTACCTAAAGCCCAGCCAATCACCTAAAGTCAACCTTCGGTTGACCTTGAGGGTTCCATAAGGGTTGGGGATGACCCTTGGGTTTGTCTTTGGGTGTTACCTTGAGTGTCTCTCtgctgtctcttt                          >  1:589845/1‑291 (MQ=255)`  
           `ataagagtcagggTCTATCCTAAGTTCTATACCTAAAGATAGGCCATCCTATCA‑GTGTCACCTAAAGATGGTCTTAGAGAGGGCCTATGGAGTTCCTATAGGGTCCTTTAAAATATACCATAAACATCTGAGTGACTATCTCACAGTGTACGGACCTAAAGTTCCCCAATAGGGGGTACCTAAAGCCCAGCCAATCACCTAAAGTCAACCTTCGGTTGACCTTGAGGGTTCCCTAAGGGTTGGGGATGACCCTTGGGTTTGTCTTTGGGTGTTACCTTGAGTGTCTCTCTG‑TGTCCCtatc                         <  1:141073/290‑4 (MQ=255)`  
              `ccTCTAATGGTCTATCCTAAGGTCTATACCTAAAGATAGGCCATCCTATCA‑GTGTCACCTAAAGAGGGTCTTAGAGAGGGCCTATGGAGTTCCTATAGGGTCCTTTAAAATATACCATAAAAATCTGAGTGACTATCTCACAGTGTACGGACCTAAAGTTCCCCCATAGGGGGTACCTAAAGCCCAGCCAATCACCTAAAGTCAACCTTCGGTTGACCTTGAGGGTTCCCTAAGGGTTGGGGATGACCCTTGGGTTTGTCTTTGGGTGTTACCTTGAGTGTCTCTCtgctgtctcttatac                       >  1:458134/1‑288 (MQ=255)`  
               `ctctAATGGTCTATCCTAAGGTCTATACCTAAAGATAGGCCATCCTATCA‑GTGTCACCTAAAGAGGGTCTTAGAGAGGGCCTATGGAGTTCCTATAGGGTCCTTTAAAATATACCATAAAAATCTGAGTTACTATCTCACAGTGTACGGACCTAAAGTTCCCCCATAGGGGGTACCTAAAGCCCAGCCAATCACCTACAGTCAACCTTCGGTTGACCTTGAGGGTTCCCTAAGGGTTGGGGATGACCCTTGGGTTTTTCTTTGGTTGTTACCTTGAGTGTCTCTCtgctgtctcttataca                      >  1:224079/1‑287 (MQ=255)`  
                 `ctAATGGTCTATCCTAAGGTCTATACCTAAAGATAGGCCATCCTATCA‑GTGTCACCTAAAGAGGGTCTTAGAGAGGGCCTATGGAGTTCCTATAGGGTCCTTTAAAATATACCATAAAAATCTGAGTGACTATCTCACAGTGTACGGACCTAAAGTTCCCCCCTAGGGGGTACCTAAAGCCCAGCCAATCACTTAAAGTCAACCTTCGGTTGACCTTGAGGGTTCCCTAAGGGTTGGGGTTGACCCTTGGGTTTGCCTTTGGGTGTTACCTTTAGTGTCTTTCtgctgtctcttataaaca                    >  1:250070/1‑285 (MQ=25)`  
                 `ctAATGGTCTATCCTAAGGTCTATACCTAAAGATAGGCCATCCTATCA‑GTGTCAACTAAAGAGGGTCTTAGAGAGGGCCTATGGAGTTCCTATCGGGTCCTTTAAAATATACCATAAAAATCTGAGTGACTATCTCACAGTGTAAGGACCTAAAGTTCCCCCATAGGGTGTACCTAAAGCCCAGCCAATCACCTAAAGTCAACCTTCGGTTGACCTTGAGGGTTACCTAAGGGTTGGGGATGACGCTTGGGGTTATCTTTGGGTGTTACCTTGAGTGTCTCTCTGCTGTCCCttatacaca                    >  1:93173/1‑293 (MQ=25)`  
                   `aaTGGTCTATCCTAAGGTCTATACAGAAAGATAGGCCATCCTATCA‑GTGTCACCTAAAGAGGGTCTTAGAGAGGGCCTATGGAGTTCCTAGAGGGTCCTTTAAAATATACCATAAAAATCTGAGTGACTATCTCACAGTGTACGGACTTAAAGTTCCCCCATAGGGGGTACCTAAAGTCCAGCAAATCACCTAAAGTCAACCTTCGGTTGACCTTGAGGGTTCCCTAAGGGTTGGGGATGACCCTTGGGTTTGTCTTTGGGTGTTACCTTGAGTGTCTCTCTG‑TGTCCCtatctgttacag                 <  1:443785/301‑12 (MQ=255)`  
                     `tGGTCTATCCTAATGTCTATACCTAAAGATAGGCCATCCTATCA‑GTGTCACCTAAAGAGTGTCTTAGAGAGGGCCTATGGAGTTCCTATAGGGTCCTTTAAAATATACCATAAAAATCTGAGTGACTATCTCACAGTGTACGGACCTAAAGTTCCCCCATAGGGGGTACCTAAAGCCCAGCCAATCACCTAAAGTCAACCTTCGGTTGACCTTGAGGGTTCCCTAAGGGTTGGGGATGACCCTTGGGTTTGTCTTTGGGTGTTACCTTGAGTGTCTCTCTG‑TGTCCCtatctgttacagtc               <  1:158996/301‑14 (MQ=255)`  
                      `ggTCTATCCTAAGGTCTATACCTAAAGATAGGCCATCCTATCA‑GTGTCACCTAAAGAGGGTCTTAGAGAGGGCCTATGGAGTTCCTATAGGGTCCTTTAAAATATACCATAAAAATCTGAGTGACTATCTCACAGTGTACGGACCTAAAGTTCCCCCATAGGGGGTACCTAAAGCCCAGCCAATCACCTAAAGTCAACCTTTGGTTGACCTTGAGGGTTCCCTAAGGGTTGGGGATGACCCTTGGGTTTGTCTTTTGGTGTTACCTTGAGTGTCTCTCtgctgtctcttatacacatctcc               >  1:135051/1‑280 (MQ=255)`  
                           `aTCCTAAGTGCTATACTTAAAGATAGGCCTTCCTATCA‑GTGTCACCTAAAGATGGTCTTAGAGAGGGCCTATGGTGTTCCTATAGGGTCCTTTAAAATATACCATAAAAATCTGAGTGACTATCTCACAGTGTACGGACCTAAAGTTCCCCCATAGGGGGTACCTAAAGCCAAGCCAATCACCTAAAGTCAACCTTCGGTTGTCCTTGAGGGTTCCCTAAGGGTTGGGGATGACCCTTGGGTTTGTCTTTGGGTGTTACCTTGAGTGTCTCTCTG‑TGTCCCtatctgttacagtctcctaa         <  1:161855/301‑20 (MQ=18)`  
                           `aTCCTAAGGTCTATACCTAAAGATAGGCCATCCTATCA‑GTGTCACCTAAAGAGGGTCTTAGAGAGGGCCTATGGAGTTCCTATAGGGTCCTTTAAAATATACCATAAAAATCTGAGTGACTATCTCACAGTGTACGGCCCTAAAGTTCCCCCATAGGGGGTACCTAAAGCCCAGCCAATCACCTAAAGTCAACCTTCGGTTGACCTTGAGGGTTCCCTAAGGGTTGGGGATGACCCTTGGGTTTGTCTTTGGGTGTTACCTTGAGTGTCTCTCtgctgtctcttatacacatctccgagcc          >  1:293940/1‑275 (MQ=255)`  
                           `aTCCTAAGGTCTATACCTAAAGATAGGCCATCCTATCA‑GTGTCACCTAAAGAGGGTCTTAGAGAGGGCCTATGGAGTTCCTATAGGGTCCTTTAAAATATACCATAAAAATCTGAGTGACTATCTCACAGTGTACGGACCTAAAGTTCCCCCATAGGGGGTACCTAAAGCCCAGCCAATCACCTATAGTCAACCTTTGGTTGACCTTGAGGGTTCCCTAAGGGTTGGGGATGATCCTTGGGTTTGTCTTTGGGTGTTACCTTGAGTGTCTCCTTGCTGTCCCttatatacatctccgaatc          >  1:77094/1‑283 (MQ=25)`  
                           `aTCCTAAGGTCTATACCTAAAGATAGGCCATCCTATCA‑GTGTCACCTAAAGAGGGTCTTAGAGAGGGCCTATGGAGTTCCTATAGGGTCCTTTAAAATATACCATAAAAATCTGAGTGACTATCTCACAGTGTACGGACCTAAAGTTCCCCCATAGGGGGTACCTAAAGCCCAGCCAATCACCTAAAGTCAACCTTCGGTTGACCTTGAGGGTTCCCTAAGGGTTGGGGCTGACCCTTGGGTTTGTCTTTGGGTGTTACCTTGAGTGTCTCTCtgctgtctcttatacacatctccgagcc          >  1:585071/1‑275 (MQ=255)`  
                           `aTCCTAAGGTCTATACCTAAAGATAGGCCATCCTATCA‑GTGTCACCTAAAGAGGGTCTTAGAGAGGGCCTATGGAGTTCCTATAGGGTCCTTTAAAATATACCATAAAAATCTGAGTGACTATCTCACAGTGTACGGACCTAAAGTTCCCCCATAGGGGGTACCTAAAGCCCAGCCAATCACCTAAAGTCAACCTTCGGTTGACCTTGAGGGTTCCCTAAGGGTTGGGGATGACCCTTGGGTTTGTCTTTGGGTGTTACCTTGAGTGTCTCTCtgctgtctcttatacacatctccgagcc          >  1:552497/1‑275 (MQ=255)`  
                           `aTCCTAAGGTCTATACCTAAAGATAGGCCATCCTATCA‑GTGTCACCTAAAGAGGGTCTTAGAGAGGGCCTATGGAGTTCCTATAGGGTCCTTTAAAATATACCATAAAAATCTGAGTGACTATCTCACAGTGTACGGACCTAAAGTTCCCCCATAGGGGGTACCTAAAGCCCAGCCAATCACCTAAAGTCAACATTCGGTTGACCTTGAGGGTTCCCTAAGGGTTGGGGATGACCCTTGGGTTTGTCTTTGGGTGTTACCTTGAGTGTCTCTCtgctttctcttatacacatctccgagcc          >  1:503251/1‑275 (MQ=255)`  
                           `aTCCTAAGGTCTATACCTAAAGATAGGCCATCCTATCA‑GTGTCACCTAAAGAGGGTCTTAGAGAGGGCCTATGGAGTTCCTATAGGGTCCTTTAAAATATACCATAAAAATCTGAGTGACTATCTCACAGTGTACGGACCTAAAGTTCCCCCATAGGGGGTACCTAAAGCCCAGCCAATAACCTAAAGTCAACATTCGGTTTAACTTTAGGGTTCCCTAAGGGTTGGGGGTGACCCTTGGGTTTGTCTTTGGGTGTTACCTTTAGTGTCTATCtgctgtcacttttacacatctactagcc          >  1:315267/1‑275 (MQ=25)`  
                           `aTCCTAAGGTCTATACCTAAAGATAGGCCATCCTATCA‑GTGTCACCTAAAGAGGGTCTTAGAGAGGGCCTATGGAGTTCCTATAGGGTCCTTTAAAATATACCATAAAAATCTGAGTGACTATACCACAGTGTACGGACCTAAAGTTCCCCCATAGGGGGCACCTAAAGCCCTGCCAATCACCTAAAGACAACCTACGGTTCACCTTGTGGGTTCCCTAAGGGTTGGGAATGACCCCTGGGGTTGTCTTTTGGTGCAACCTTGAGTGTCCATCtg‑tcgtctcttatactgaccttggagcc         >  1:433119/1‑276 (MQ=25)`  
                                 `aGGTCTATACCTAAAGATAGGCCATCCTATCA‑GTGTCACCTAAAGAGGGTCTTAGAGAGGGCCTATGGAGTTCCTATAGGGTCCTTTAAAATATACCATAAAAATCTGAGTGACTATCTCACAGTGTACGGACCTAAAGTTCCCCCATAGGGGGTACCTAAAGCCCAGCCAATCACCTAAAGTCAACCTTAGGTTGCCCTTGAGGGTTCCCTAAGGGATGGGGATGACCCTTGGGTTTGTATTTGGTTGTTACTTTGATTGTCTCTCTGCTGTcctttatacatctctcccagccaccgaa    >  1:88558/1‑275 (MQ=21)`  
                                   `gTCTATACCTAAAGATAGGCCATCCTATCA‑GTGTCACCTAAAGAGGGTCTTAGAGAGGGCCTATGGAGTTCCTATAGGGTCCTTTAAAATATACCATAAAAATCTGAGTGACTATCTCACAGTGTACGGACCTAAAGTTTCTCCCTAGGGGGTACATAAAGCCCAGCCAATCACCTAAAGTCAACCTTCGGTTGATCTTGAGGGTTCCCTAAGGGATGGGGATGACCCTTGGGTTTGTCTTTGGGTGTTACCTTGAGTGACTGTCTGCTGTCCCttatacacatctccgagcccacgagac  >  1:240216/1‑275 (MQ=21)`  

                                                                                                                                                                                                                                                                                                               `|`                                   
`ATACCCATAAAGAGCCTCTAATGGTCTATCCTAAGGTCTATACCTAAAGATAGGCCATCCTATCA‑GTGTCACCTAAAGAGGGTCTTAGAGAGGGCCTATGGAGTTCCTATAGGGTCCTTTAAAATATACCATAAAAATCTGAGTGACTATCTCACAGTGTACGGACCTAAAGTTCCCCCATAGGGGGTACCTAAAGCCCAGCCAATCACCTAAAGTCAACCTTCGGTTGACCTTGAGGGTTCCCTAAGGGTTGGGGATGACCCTTGGGTTTGTCTTTGGGTGTTACCTTGAGTGTCTCTCTG‑TGTCCCT‑‑‑‑‑‑‑‑‑‑‑‑‑‑‑‑‑‑‑‑‑‑‑‑‑‑  >  NC_001604.1/39629‑39937`

  

Alignment Legend

Aligned base mismatch/match (shaded by quality score): `ATCG/ATCG` `< 3 ≤` `ATCG/ATCG` `< 10 ≤` `ATCG/ATCG` `< 20 ≤` `ATCG/ATCG` `< 34 ≤` `ATCG/ATCG` `< 38 ≤` `ATCG/ATCG`

Unaligned base: `atcg`    Masked matching base: `atcg`    Alignment gap: `‑`    Deleted base: `‑`
